# When Skin-Limited Langerhans Cell Histiocytosis Becomes Life-Threatening: Severe Treatment-Related Morbidity in a Prematurely Born Infant—Case Report

**DOI:** 10.3390/reports9030264

**Published:** 2026-08-10

**Authors:** Nusa Matijasic Stjepovic, Izabela Kranjcec, Aleksandra Bonevski

**Affiliations:** 1 Department of Oncology and Hematology, Children’s Hospital Zagreb, 10000 Zagreb, Croatia; nusa.matijasic@gmail.com (N.M.S.);; 2 Faculty of Medicine, University of Rijeka, 51000 Rijeka, Croatia; 3 Department of Pediatrics, University North, 42000 Varazdin, Croatia

**Keywords:** Langerhans cell histiocytosis, cutaneous Langerhans cell histiocytosis, pediatric histiocytosis, LCH-IV trial, LCH treatment side effects

## Abstract

**Background and Clinical Significance:** Skin-limited Langerhans cell histiocytosis (LCH) is a clinically heterogeneous disease, ranging from self-healing forms to fulminant multi-organ failure, the latter being more often described in infants, especially preterm neonates. The optimal therapy for cutaneous LCH remains controversial; the possibilities vary from a watchful waiting approach to systemic chemotherapy. **Case Presentation:** This case report describes an exceptionally rare and clinically challenging course of skin-limited LCH in a prematurely born infant treated at the Department of Oncology and Hematology, Children’s Hospital Zagreb, Croatia. At presentation, the patient exhibited several features suggestive of aggressive disease biology. However, therapeutic decision-making was complicated by extreme prematurity and young age, both of which significantly increased vulnerability to treatment-related toxicity. Following failure of topical therapy, systemic treatment was initiated according to the LCH-IV trial, primarily due to concerns regarding potential evolution into multisystem LCH. During treatment, the patient developed multiple life-threatening complications, namely severe infections (*Staphylococcus aureus* endocarditis, *Pneumocystis jirovecii* pneumonia, and *Enterobacter cloacae* sepsis), aggravated by secondary hypogammaglobulinemia, neutropenia, and iatrogenic adrenal insufficiency. **Conclusions:** The varied nature of cutaneous LCH underscores the necessity for a tailored treatment approach. When deciding on the treatment modality, clinicians should weigh the benefits of aggressive therapies, ensuring better disease control, against the potential for severe adverse effects, particularly in young, fragile infants with immature immunity.

## 1. Introduction and Clinical Significance

Langerhans cell histiocytosis (LCH) is a clinically heterogeneous disease caused by abnormal proliferation of CD1a+/CD207+ dendritic cells, and their accumulation in various tissues and organs [1,2,3]. The annual incidence rate in children younger than 15 years is 4.6 per one million children, with a significantly higher rate in infants (≅15/10^6^) [4,5]. The underlying molecular pathogenesis is driven by abnormal activation of the mitogen-activated protein kinase (MAPK) signaling pathway, with the most prevalent alterations including *BRAFV600E* and *MAP2K1* mutations [3,6,7]. This rare and enigmatic entity may range from a self-limiting condition to a highly aggressive malignancy. Risk organ involvement (liver, spleen and bone marrow) is associated with worse prognosis [3]. Due to the broad spectrum of clinical presentations, often mimicking other disorders, with overlapping histopathological features and an unpredictable disease course, LCH poses a unique diagnostic, as well as therapeutic challenge.

The disease is mainly unifocal, with multisystem forms usually seen in infants [4]. Skin is the second most frequently affected organ, following the skeleton. However, in children younger than two, skin is the number one target organ. Although LCH can masquerade practically any dermatologic condition, seborrheic dermatitis and eczematous eruptions on the scalp and trunk are the most common morphologic findings in infants [4,8]. Unfortunately, skin lesions at a young age often represent only an initial manifestation of a more severe, extensive disease [8,9,10,11].

We herein describe a case of a premature male infant diagnosed with cutaneous LCH, who achieved complete remission following the introduction of systemic therapy, during which he suffered from numerous, nearly fatal complications. This patient experienced one of the most challenging cases of cutaneous LCH managed at our institution. His case raised a lot of important questions, faced us with some difficult decisions and in the end left us with a few valuable lessons learned. Treatment of skin-limited LCH remains controversial. The decision to start systemic chemotherapy was especially challenging in this situation, as the fragile infant’s body with immature defense mechanisms was specifically prone to treatment-related toxicity. However, this was the risk we decided to take—the course of LCH is unpredictable, and the patient had various factors in favor of a more aggressive disease biology.

## 2. Case Presentation

In May of 2021, a six-month-old male infant was admitted to the Department of Oncology and Hematology, Children’s Hospital Zagreb, Croatia for further evaluation of a diffuse erythematous papular rash, suspicious for cutaneous LCH. He was a prematurely born child, delivered through an emergency cesarean section at 28 weeks of gestation from a monochorionic monoamniotic pregnancy, with the development of feto-fetal transfusion syndrome, the patient being the donor twin. Due to numerous complications of prematurity, including respiratory distress syndrome requiring noninvasive support, atrial septal defect, jaundice, failure to thrive, grade I intraventricular hemorrhage and generalized hypotonia, he was intensively monitored and treated at the neonatology ward of another hospital during the first two months of his life. He was discharged home in good general condition, without the requirement of additional oxygen, and with a satisfactory daily intake of milk formula.

The itchy rash first erupted when the boy was four months old. Initially, scabies was considered in the differential diagnosis; however, several clinical and epidemiological findings argued against this diagnosis. The patient’s epidemiological history was unremarkable, with no household members or close contacts exhibiting similar symptoms. As a prematurely born infant, he was cared for in a protected environment with minimal exposure to other children, apart from his twin brother. The patient was evaluated independently by three experienced dermatologists—two working in a university clinical hospital and one in private practice. None identified dermoscopic features suggestive of scabies, and repeated skin scrapings were consistently negative for *Sarcoptes scabiei* mites and eggs. Nevertheless, as the cutaneous lesions continued to progress and the diagnosis remained uncertain, empirical treatment with permethrin was administered with no clinical improvement, further reducing the likelihood of scabies.

**Figure 1:**
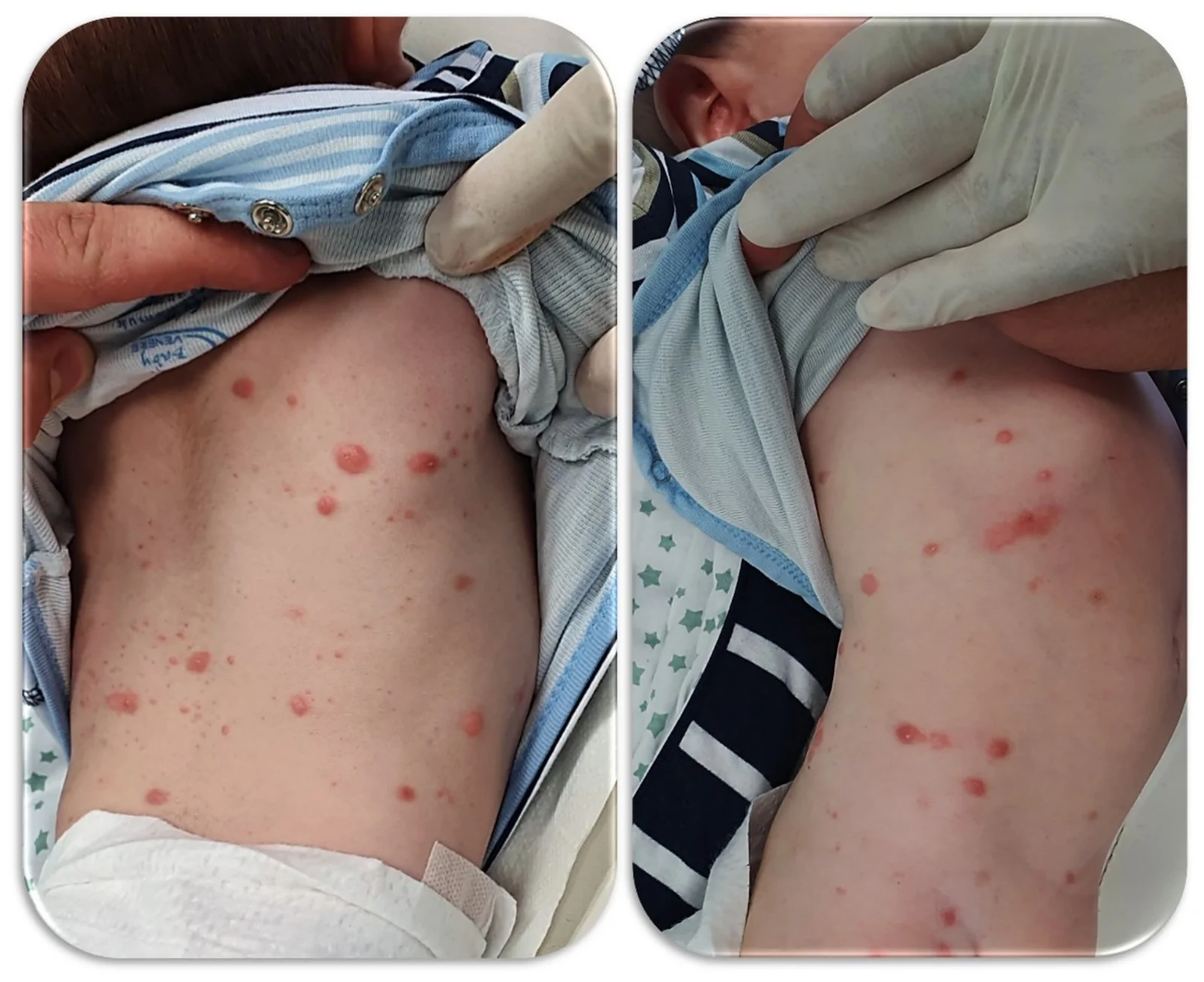
The photographs of the patient taken at admission showing the young patient’s back and trunk which were covered in erythematous papules up to 1 cm in size.

Upon admission to our Department, the patient had densely disseminated infiltrated erythematous papules up to 1 cm in size, located on the scalp, trunk and extremities, visible in Figure 1. No other signs nor symptoms, possibly indicating multisystem LCH, were noted. The child was cardiorespiratory compensated, afebrile, without lymphadenopathy, hepatosplenomegaly, bone or soft tissue swelling. Moreover, he did not have polydipsia, polyuria, diarrhea, nor any new neurological deficits. His family history for malignancies was unremarkable.

**Figure 2:**
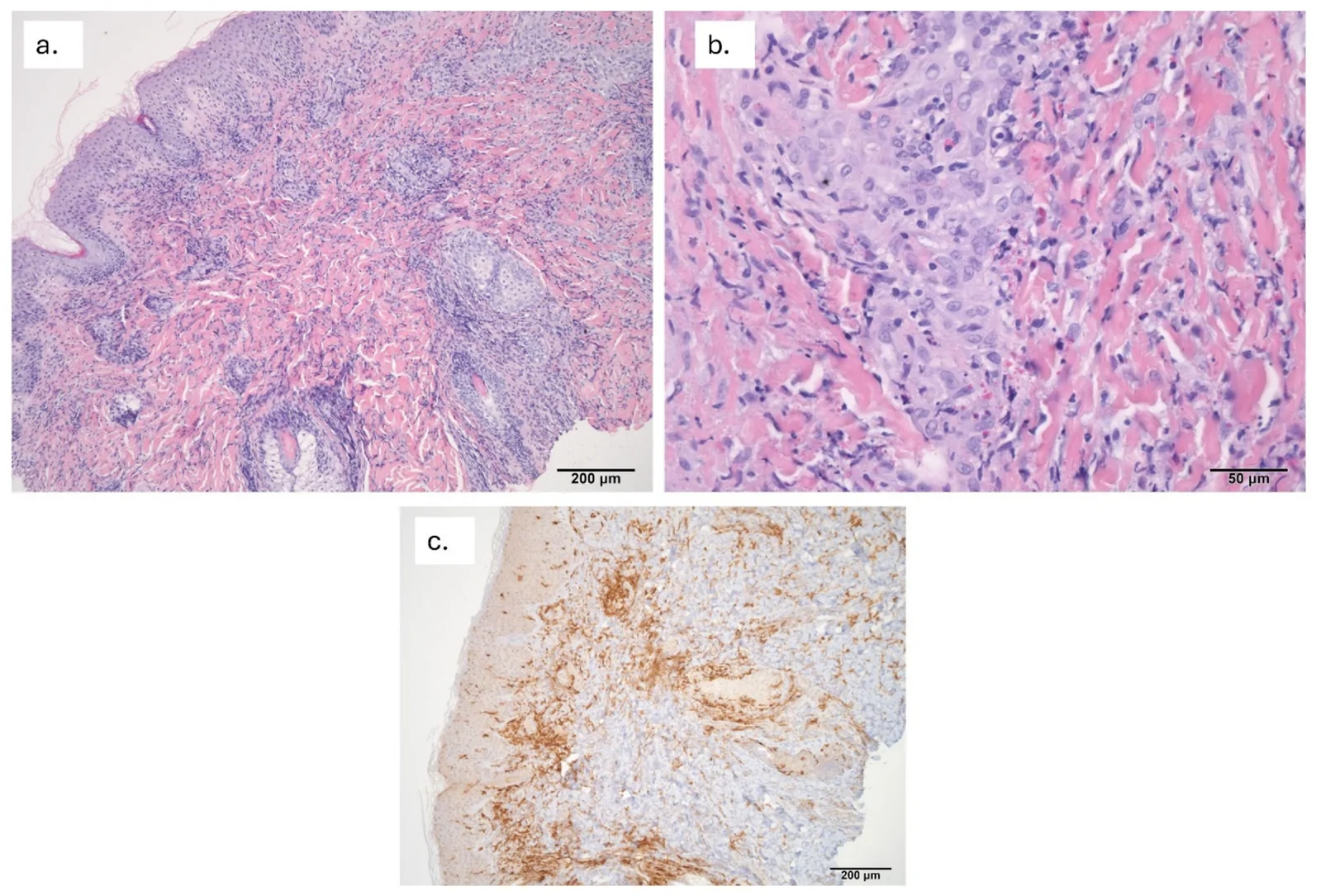
Microscopic view of skin biopsy. (**a**) Dermis is infiltrated with Langerhans cells and inflammatory infiltrates (hematoxylin–eosin stain × 40). (**b**) Higher magnification showing Langerhans cells with abundant, pale eosinophilic cytoplasm, irregular and elongated nuclei with prominent nuclear grooves and folds, fine chromatin and indistinct nucleoli, surrounded with few lymphocytes and eosinophils (hematoxylin–eosin stain × 200). (**c**) Immunohistochemical staining showing positive CD1a reactivity (CD1a × 100).

Histopathological analysis of the lesional skin tissue, acquired through punch biopsy, set the diagnosis of LCH—dermal clusters of large oval cells with pale to eosinophilic cytoplasm and irregular and elongated nuclei were described, with clonal cells showing characteristic diffuse positivity for CD1a on immunohistochemical staining (Figure 2).

Furthermore, molecular analysis of the formalin-fixed paraffin-embedded tissue specimen identified *BRAF* gene mutation—*V600E*—the most typically encountered genetic alteration in LCH. The analysis was performed using allele-specific real-time polymerase chain reaction (PCR) with the Idylla *BRAF* Mutation Assay. An extensive work-up was conducted, excluding dissemination and risk organ involvement. Findings of relevant laboratory tests and medical imaging are summarized in Table 1.

At first, topical treatment was used to treat skin lesions, but without any improvement; instead, the rash progressed. Some of the initial erythematous papules gradually increased in size and became more prominent, while new lesions continued to emerge, mainly on the trunk and in the diaper area. In several areas, adjacent papules coalesced to form larger erythematous plaques, resulting in more extensive skin involvement. Overall, the rash exhibited both an increase in the number of lesions and progression in their size and distribution, indicating ongoing disease activity despite topical treatment. Finally, systemic chemotherapy was initiated, according to the currently ongoing international trial of the Histiocyte Society—LCH-IV (International Collaborative Treatment Protocol for Children and Adolescents with LCH). The first-line therapy consisted of a combination of oral prednisone and vinblastine injections. Due to the expected iatrogenic immunosuppression caused by prolonged corticosteroid use and chemotherapeutics, pharmaceutical prophylaxis against *Pneumocystis jirovecii* (*P. jirovecii*) was introduced. In line with the treatment protocol the drug of choice was oral liquid formulation of trimethoprim/sulfamethoxazole (TMP/SMX), 5 mg/kg/day of trimethoprim, divided into two daily doses, given three times per week (every second day) [12].

**Figure 3:**
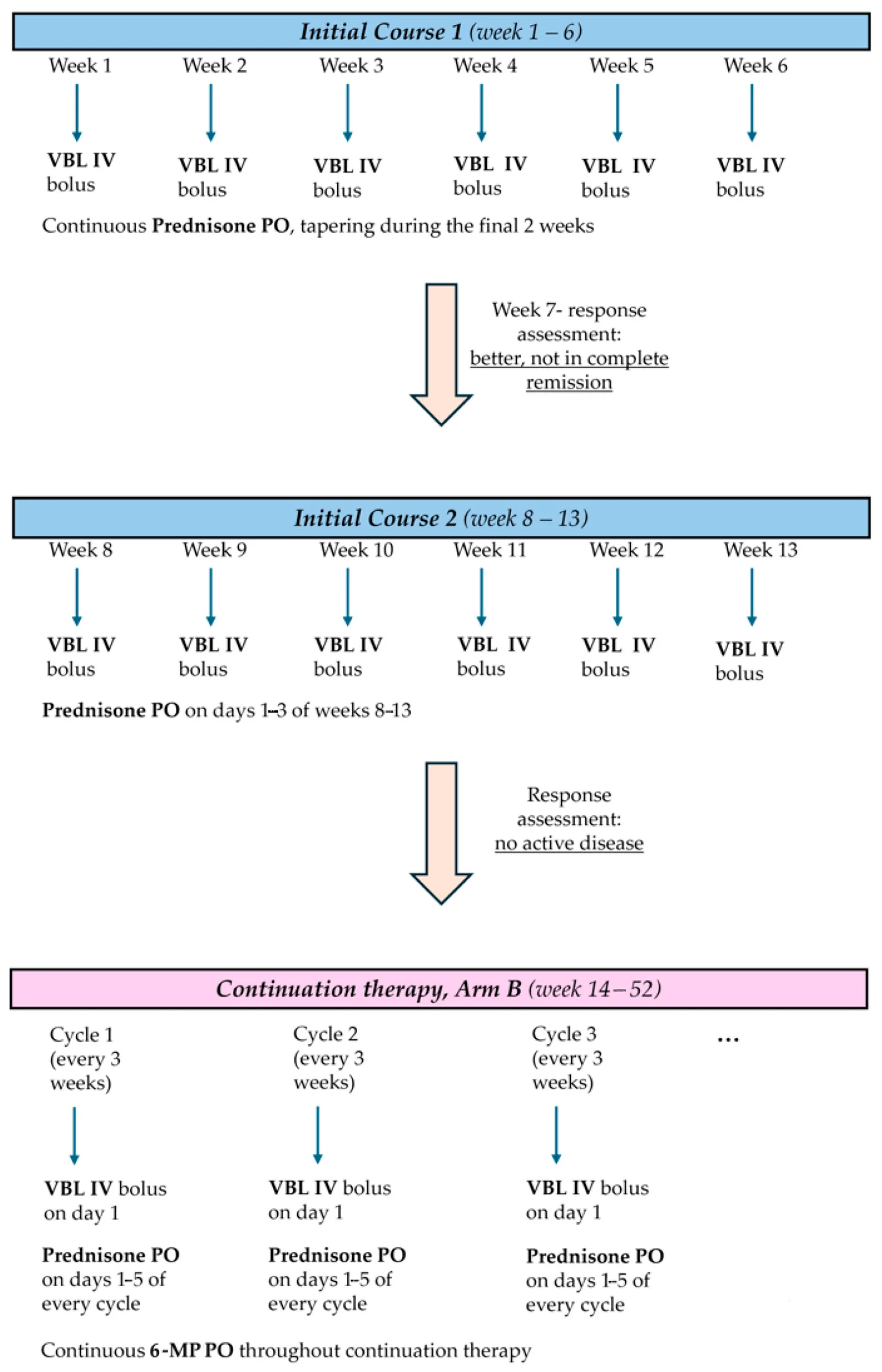
The figure depicts therapy scheme the patient received, according to the LCH-IV trial. VBL—Vinblastine, IV—Intravenous, PO—Peroral, 6-MP—Mercaptopurine.

Surprisingly, after the initial improvement, during induction therapy the skin status once again deteriorated, and so did the pruritus and nighttime fussiness. Soon the mother developed an itchy rash. It was brought to attention that the patient potentially got in contact with scabies in domestic environment through young family members attending a day care facility where the infection was reported. At this point, *Sarcoptes scabiei* infection was confirmed by cytological examination of the affected skin. Local application of an antiparasitic drug relieved the symptoms, and induction therapy was continued according to the protocol. By the end of Initial Course 1, partial remission was obtained, so Initial Course 2 followed, altogether lasting for 13 weeks (in between courses one week was spent on response assessment). Figure 3 depicts the treatment plan according to the LCH-IV trial.

During the induction phase, the infant suffered from catheter-related sepsis caused by *Staphylococcus aureus*, needing emergency central venous line removal. Due to prolonged fever with positive echocardiographic signs of infective endocarditis, intravenous anti-staphylococcal penicillin was administered for four weeks, with excellent treatment response.

**Figure 4:**
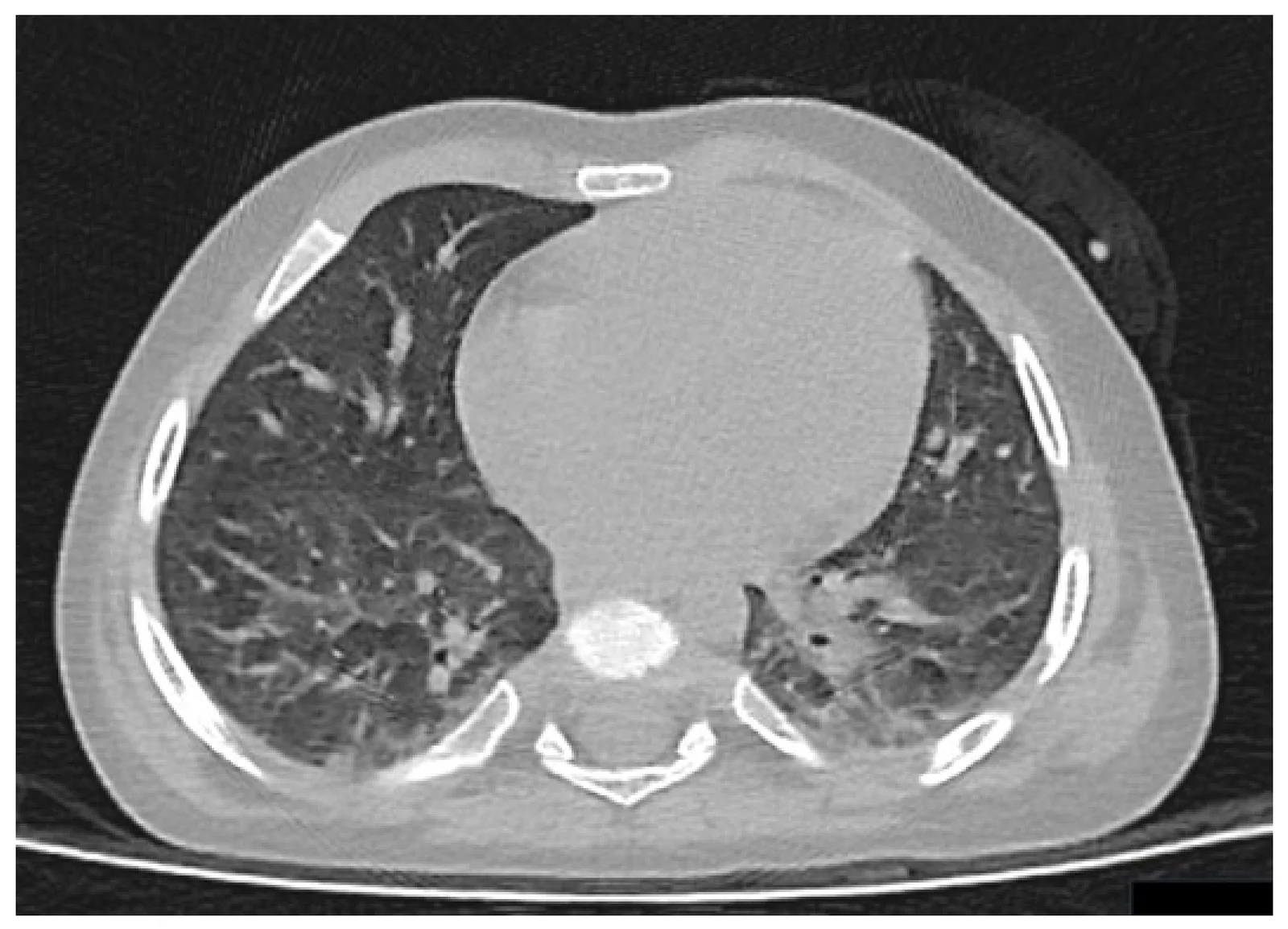
MSCT of the lungs showing ground glass opacities.

In the Initial Course 2, the patient’s condition deteriorated once again in form of tachypnea and hypoxemia, without other clinical, laboratory or X-ray signs of pneumonia. In addition, the infant had failure to thrive, abundant regurgitation and often vomited. Symptomatic microaspirations of the gastric content were believed to be the culprit, and 24 h esophageal pH-monitoring with impedance confirmed pathological reflux. Despite strict anti-reflux measures, tachypnea worsened rapidly. Finally, multislice computed tomography (MSCT) of the lungs revealed bilateral ground glass opacities, dominantly in the lower lobes, described in rare cases of childhood pulmonary histiocytosis [13,14]. However, the diagnosis was excluded by immunohistochemical analysis of the bronchoalveolar lavage (BAL) content which demonstrated less than 5% of CD1a+ cells—a diagnostic criterion for lung LCH [12,14]. PCR of the BAL sample identified *P. jirovecii* as the causative agent, even though antimicrobial prophylaxis was introduced simultaneously with chemotherapy. Extensive immunological work-up (serum immunoglobulin concentrations, flow-cytometry-based immunophenotyping of peripheral blood lymphocytes, respiratory burst assay, T-cell proliferation test to mitogens, thymic ultrasound and human immunodeficiency virus (HIV) serology) ruled out severe congenital immunodeficiency disorders and HIV infection. A three-week-long therapy with intravenous TMP/SMX led to complete clinical and radiological lung recovery. The dosing regimen consisted of 20 mg/kg/day of the trimethoprim component, divided into three equal doses, each dose given every eight hours. Since vomiting had been significantly reduced on anti-reflux measures, including domperidone, and the mother was once again educated on the importance of regular TMP/SMX administration, prophylaxis was continued with oral TMP/SMX. Figure 4 shows MSCT images of the lungs obtained during respiratory failure.

By the end of Initial Course 2, the patient was in complete remission. Continuation therapy consisted of vinblastine, prednisone and 6-mercaptopurine, as shown in Figure 2.

At the start of the second continuation course, shortly upon injecting vinblastine through port catheter, the infant developed signs of a bloodstream infection, proven to be caused by extended spectrum β-lactamase (ESBL) producing *Enterobacter cloacae*. Given the prompt clinical response to carbapenems and antibiotic lock therapy, the intraluminal central venous access device was salvaged. Unfortunately, when the port catheter was used for drug administration in the following cycle, the patient fell into a septic shock, caused by the same agent (*Enterobacter cloacae* ESBL), requiring intensive care support measures. He depended on high intravenous hydrocortisone doses due to iatrogenic adrenal insufficiency. This time, the catheter was removed, and the two last courses of chemotherapy were administered through a peripheral vein, luckily without unwanted events.

Throughout the intensive treatment the patient was in the care of a multidisciplinary team formed of a network of pediatric specialists including oncologists, dermatologists, neurologists, gastroenterologists, pulmonologists, immunologists, endocrinologists, radiologists, physical medicine specialists and nutritionists, to provide the best possible, individualized and holistic approach. Four years after treatment cessation the boy is in complete remission, without long term LCH or treatment sequalae.

**Figure 5:**
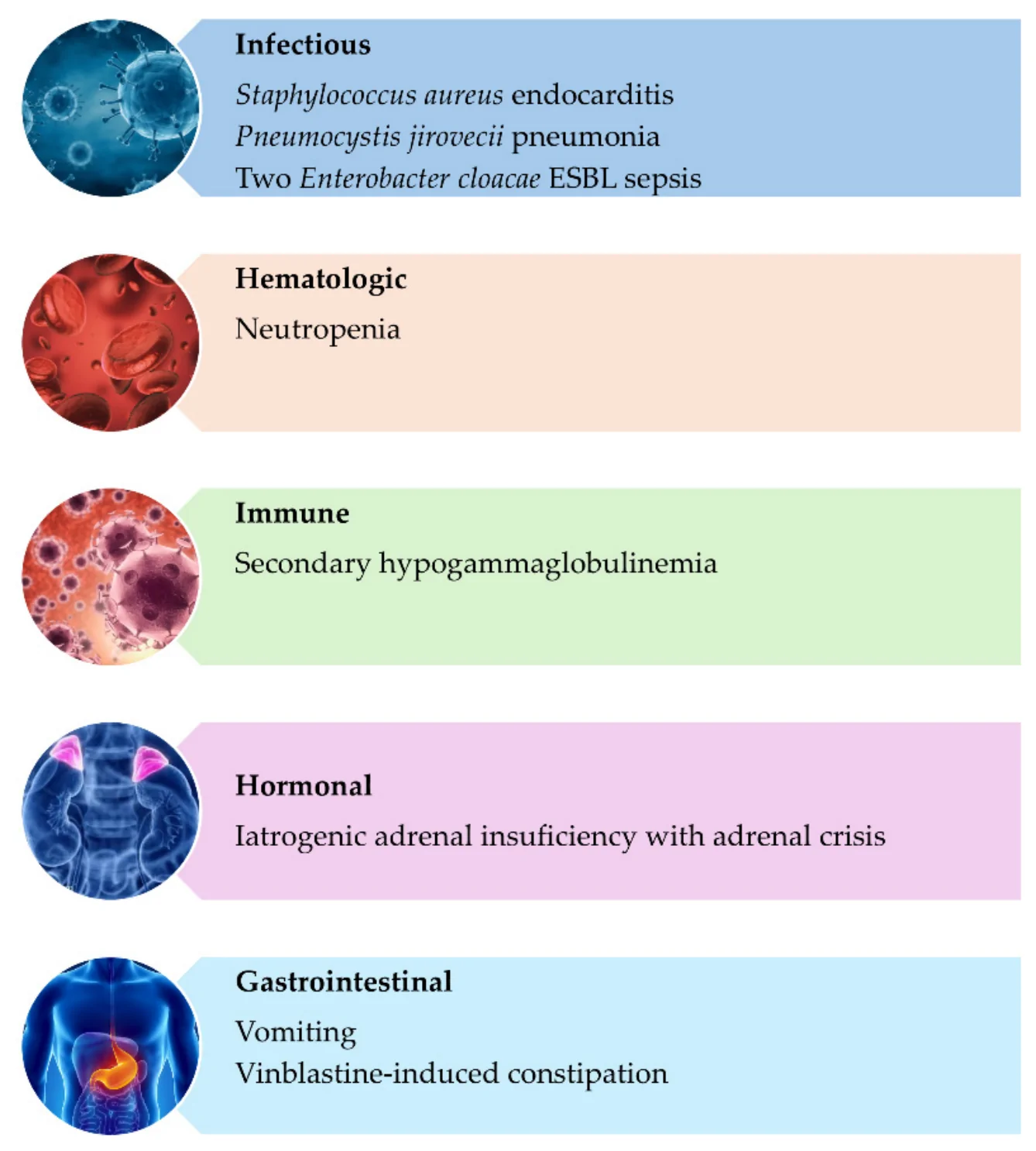
Treatment-related complications which developed in the patient.

Figure 5 contains visual representation of the main treatment-related toxicities which developed in the patient during systematic therapy of cutaneous LCH.

## 3. Discussion

The above-described patient had one of the most complex clinical courses of skin-limited LCH managed in our department. His case of LCH, being a rare condition itself, in addition to other aggravating circumstances, raised many questions during the diagnosis process, as well as the treatment. The following text addresses major concerns regarding his case, with the idea of explaining the chosen approach, hopefully facilitating decision-making for clinicians encountering similar pediatric LCH patients in the future.

### 3.1. When to Suspect Cutaneous LCH in a Young Infant?

Histiocytic skin lesions can resemble almost any skin morbidity. Setting the diagnosis in young infants can be challenging, considering the frequency and variety of rashes (mainly infectious) and numerous other dermatologic conditions. Most commonly, cutaneous LCH presents as erythematous papules dispersed on the scalp and trunk, as seen in our patient, but intriguing cases of flexural involvement with fissures, moist scales, as well as hypopigmented and depigmented macules have also been reported [10,11]. In fact, although skin LCH most often resembles seborrheic eczema, literature reports unravel cases of LCH in infants mistakenly treated as candidiasis [15], congenital herpes simplex virus infection [16], and persistent diaper dermatitis [17]. It may even appear as “blueberry muffin” syndrome, connected to a variety of differential diagnoses such as infections (rubella, toxoplasmosis, cytomegalovirus, parvovirus B19, syphilis…), extramedullary hematopoiesis or certain types of cancer (leukemia, neuroblastoma, rhabdomyosarcoma) [18].

In our patient, clinical presentation of cutaneous LCH was typical, both regarding morphology and distribution of skin lesions. Yet, LCH being a rare disease, scabies was first suspected, due to characteristic appearance of the rash, accompanied by pruritus. We advise performing a biopsy of any persistent skin lesion of uncertain etiology. As in confirming the primary diagnosis, in cases of unexplained deterioration of cutaneous LCH during standard treatment, further cytological or histological analysis should be made, to rule out other possible conditions (like scabies in our case) [12,19].

### 3.2. Once the Diagnosis of LCH Is Made, What Should Be the Extent of the Conducted Baseline Work-Up?

LCH-IV protocol clearly outlines mandatory baseline work-up. Additional diagnostic steps are taken in exceptional circumstances, such as bone marrow analysis in the presence of multilineage or persistent cytopenia [12]. Even though our patient had no laboratory signs of hematopoietic system infiltration at the time of diagnosis, we decided to perform marrow aspiration to exclude the possibility of yet asymptomatic involvement. Although this intervention is not supported by current international trial, it was carefully considered and deemed necessary and justified in this case based on our institutional experience with the unpredictable progression of skin-limited LCH to multisystem disease in infants. While it is well known that forms of self-healing skin histiocytic disease exist, it is not uncommon in infants that LCH develops into aggressive and fulminant multisystem illness [20,21]. Another rationale for bone marrow sampling in infants could be to distinguish histiocytic spread to the marrow from the physiologic and nutritional iron deficiency anemia frequently reported in the first few months of life [22,23]. It is probable that in some young infants (though not in our case) physiologic anemia timely overlaps with the diagnosis of LCH, causing confusion for the leading physician, and warranting bone marrow analysis to exclude hematopoietic involvement.

Moreover, the patient underwent magnetic resonance imaging (MRI) of the brain, which is usually done only on specific occasions—bone lesions at the skull base, neurologic and endocrine abnormalities, aural discharge or hearing impairment [12]. Despite the absence of new neurologic deficits at the time of diagnosis, the patient’s neurodevelopment was delayed due to prematurity, and neurological status was difficult to objectify. Baseline brain MRI served to obtain an insight into the child’s central nervous system (CNS), to not overlook disease-related tumors or radiological neurodegeneration.

In conclusion, when performing initial evaluation, we advocate an individualized approach, considering all the patient’s characteristics and possible comorbidities.

### 3.3. How Do We Treat Isolated Cutaneous LCH in Infants?

As previously mentioned, pediatric patients with single system LCH are traditionally considered to have excellent prognosis, even without systemic therapy. Treatment of isolated cutaneous LCH is controversial, systemic treatment should be considered in infants who fail to respond to topical drugs [12].

Cutaneous LCH, compared to other localized forms, has a higher tendency for dissemination, especially in young infants [24,25]. Additionally, skin LCH has the potential for multiple reactivations, and progression to a chronic, refractory illness [24]. A small, but important and quite worrisome retrospective study, conducted on 22 infants with cutaneous LCH, treated during a 21-year-long period in The Hospital for Sick Children, Toronto, Canada, revealed that nearly half (N = 10, 45%) of the subjects had multisystem disease at diagnosis. Out of the remaining 12 with exclusively skin affected at diagnosis, four patients (33%) eventually progressed to multisystem disease. Moreover, 11 of the 14 (79%) patients with disease spread had a risk of organ involvement, and the mortality rate was 50% [25]. By contrast, more recent data on skin-limited LCH in childhood shows optimistic results—progression to multisystem disease is strikingly less frequent than previously reported. This may result from a more accessible pediatric subspeciality care, with higher referral rates ensuring recognition of many self-resolving skin presentations, which historically went unrecognized [26].

Limited experience based on a few cases described in the literature demonstrated that preterm neonates have a particularly poor prognosis of cutaneous LCH, the cause of which remains unclear [27,28,29,30,31,32]. Skin lesions in premature babies can be impressive, resembling “blueberry muffin rash” or even diffuse burns [27,28]. Unfortunately, in these patients LCH progressed to multi-organ failure and death within hours or days. However, in all of them, rash was congenital, which differs from our patient [27,28,29,30,31,32].

Given the young age, prematurity, unresponsiveness to topical treatment and a reasonable fear of disease progression, we have decided to introduce systemic chemotherapy in this patient, despite possible side effects. An additional risk factor in his case was the presence of *BRAFV600E* mutation, associated with more aggressive features [33,34]. Patients with the detected mutation are candidates for targeted therapy. Although vemurafenib, a selective *BRAF* inhibitor, usually leads to dramatic improvement, discontinuation of the medicine often results in relapses [35,36]. Since our patient showed an excellent response to first-line systemic therapy, no other agents were introduced.

### 3.4. How Do We Explain the Occurrence of Numerous Treatment-Related Complications in This Patient, and What Can We Do to Minimize the Risk of Serious Adverse Events in the Future?

The primal concern in our patient was a series of life-threatening infections, attributed to several complementary factors including immature immunity, secondary hypogammaglobulinemia, neutropenia, high-dose corticosteroids, repetitive invasive procedures, and bacterial biofilm formation on the implanted intraluminal device.

Even healthy newborns and young infants are highly susceptible to infections, mostly due to insufficient and inappropriate immunity [37]. Preterm neonates have particularly deficient innate and adaptive immune responses due to reduced immunoglobulin levels, inadequate opsonization and phagocytosis [38]. At one point, our patient had secondary hypogammaglobulinemia and received intravenous immunoglobulins. Due to treatment-induced neutropenia, granulocyte colony-stimulating factor (G-CSF) was administered.

Iatrogenic adrenal insufficiency additionally contributed to the fulminant course of *Enterobacter cloacae* sepsis. The condition is usually described in pediatric patients treated for acute lymphoblastic leukemia during the induction phase. High-dose steroids trick the hypothalamic–pituitary–adrenal axis into believing that the body has sufficient cortisol, leading to temporary inactivation of adrenal glands, which is specifically dangerous in situations of physiological stress. Glucocorticoid replacement therapy during stressful periods should be considered to minimize life-threatening complications [39,40,41].

Catheter-related bloodstream infections are a major problem for all patients with childhood malignancies or other conditions requiring central venous access devices. The formation of bacterial biofilm leads to repetitive bacteremia. The extracellular matrix protects the bacteria from host immune response system and antibacterial drugs [42,43]. Antibiotic lock therapy, signifying installation of high-dose antimicrobials into the device lumen to fight biofilm bacteria, in conjunction to systemic antibiotics, enables catheter retention in a considerable portion of cases [44]. In our situation, the decision to attempt catheter salvage in the first episode of *ESBL Enterobacter* infection was based on clinical judgment—the patient showed almost immediate drastic improvement on antimicrobial treatment. Removal and replacement of port-a-cath meant more exposure to anesthesia toxicity and posed a technical difficulty as the anatomy of a small infant makes guiding the new catheter into the correct position more difficult [45].

Another unfortunate event in our patient was the development of *P. jirovecii* pneumonia, despite prophylaxis with TMP/SMX. As already mentioned, the patient had pathologic gastrointestinal reflux, with abundant regurgitation and often vomited, so compliance remained questionable. Explaining the importance of adherence to TMP/SMX to patients and/or their parents is of utmost importance. In circumstances of TMP/SMX intolerance, safe alternatives are inhaled pentamidine, dapsone and atovaquone. Since first-line chemoprophylaxis is associated with the lowest risk of developing *P. jirovecii* pneumonia, efforts should be made to rechallenge with TMP/SMX whenever possible, as accomplished in our patient [46].

## 4. Conclusions

The varied nature of skin-limited LCH, ranging from self-healing to severe multi-organ failure disease, underscores the necessity for a tailored treatment approach. To our knowledge, the reported case presents one of the most complex clinical courses of cutaneous LCH, treated with systemic therapy. We emphasize the importance of careful consideration when selecting treatment modalities, particularly in vulnerable populations such as infants with immature immunity, prone to life-threatening infections. Ultimately, clinicians must weigh the benefits of aggressive therapies, ensuring better disease control, against the potential for severe adverse effects.

## Figures and Tables

**Table 1:** The table summarizes results of the baseline work-up performed to evaluate disease spread.

Diagnostic Test	Result	Reference Range for Age	Rationale for the Diagnostic Test and Explanation
Complete blood count	Hb 126 g/L E 4.32 × 10^12^/L L 13.18 × 10^9^/L Tr 551 × 10^9^/L	Hb 109–138 g/L E 4.00–5.00 × 10^12^/L L 6.00–16.00 × 10^9^/L Tr 150–450 × 10^9^/L	Mandatory work-up; no cytopenia detected
Surrogate markers of inflammation	ESR 5 mm/h Ferritin 31.0 µg/L	ESR 0–20 mm/h Ferritin 13.3–192.0 µg/L	Mandatory work-up; values are not increased as it would be expected in multisystem disease, due to widespread inflammation
Biochemistry	TP 68 g/L Albumin 43 g/L Fibrinogen 2.7. g/L Bilirubin 6 µmol/L ALT 32 U/L AST 39 U/L ALP 305 U/L γGT 24 U/L BUN 2.2 mmol/L Creatinine 22 µmol/L Na 139 mmol/L K 5.1 mmol/L	TP 55–80 g/L Albumin 28–48 g/L Fibrinogen 1.6–4 g/L Bilirubin <20 µmol/L ALT 11–46 U/L AST 26–75 U/L ALP 25–500 U/L γGT 1–39 U/L BUN 1.0–7.5 mmol/L Creatinine 14–34 µmol/L Na 134–142 mmol/L K 3.0–7.0 mmol/L	Mandatory work-up; values within the reference range for age indicating normal hepatic and renal function, without laboratory signs of hepatic lesion or cholestasis
Specific gravity and osmolality of the early morning sample urine	Gravity 1.010 kg/L Osmolality—was not obtained on initial evaluation due to technical reasons	Gravity 1.005–1.025 kg/L	Mandatory work-up; normal urine gravity and inexistence of polyuria/polydipsia exclude diabetes insipidus
Abdominal ultrasound	Size and structure of the liver and spleen were normal for age	/	Mandatory work-up; no sonographic signs of risk organ involvement
Chest radiograph	Clear, air-filled lungs without abnormal masses, fluids, or consolidations	/	Mandatory work-up; no radiographic signs of lung involvement
Bone marrow aspirate analysis	Normocellular marrow, without atypical CD1a positive cells	/	Additional work-up; analysis was performed to exclude disseminated disease; the patient did not have cytopenia, but had other risk factors featuring aggressive disease biology
Brain MRI	Delayed myelination, but without signs of CNS LCH	/	Additional work-up, normal finding excluded the existence of tumorous lesions or LCH neurodegeneration, delayed myelination was a consequence of prematurity

Hb—Hemoglobin, E—Erythrocytes, L—Leukocytes, Tr—Thrombocytes, ESR—Erythrocyte sedimentation rate, TP—Total protein, ALT—Alanine aminotransferase, AST—Aspartate aminotransferase, ALP—Alkaline phosphatase, γGT—Gamma-glutamyl transferase, BUN—Blood urea nitrogen, Na—Sodium, K—Potassium, MRI—Magnetic resonance imaging, CNS—Central nervous system, LCH—Langerhans cell histiocytosis.

## Data Availability

The data supporting the findings of this case report are available from the corresponding author upon reasonable request, in accordance with privacy and ethical restrictions.

## Abbreviations

The following abbreviations are used in this manuscript, in order of appearance:

LCH	Langerhans cell histiocytosis
MAPK	Mitogen-activated protein kinase
PCR	Polymerase chain reaction
Hb	Hemoglobin
E	Erythrocytes
L	Leukocytes
Tr	Thrombocytes
ESR	Erythrocyte sedimentation rate
TP	Total protein
ALT	Alanine aminotransferase
AST	Aspartate aminotransferase
ALP	Alkaline phosphatase
γGT	Gamma-glutamyl transferase
BUN	Blood urea nitrogen
Na	Sodium
K	Potassium
MRI	Magnetic resonance imaging
CNS	Central nervous system
*Pneumocystis jirovecii*	*P. jirovecii*
TMP/SMX	Trimethoprim/sulfamethoxazole
VBL	Vinblastine
IV	Intravenous
PO	Peroral
6-MP	6-Mercaptopurine
MSCT	Multislice computed tomography
BAL	Bronchoalveolar lavage
HIV	Human immunodeficiency virus
ESBL	Extended spectrum β-lactamase
G-CSF	Granulocyte colony-stimulating factor
